# Older adults’ perceptions about meat consumption: a qualitative study in Gasabo district, Kigali, Rwanda

**DOI:** 10.1186/s12889-024-19038-z

**Published:** 2024-06-05

**Authors:** Theogene Habumugisha, Inger E. Måren, Eric Matsiko, Matthias Kaiser, Jutta Dierkes, Ingunn M.S. Engebretsen

**Affiliations:** 1 Centre for International Health, Department of Global Public Health and Primary Care, University of Bergen, Årstadveien 21, Bergen, 5009 Norway; 2 Department of Biological Sciences, University of Bergen, Bergen, Norway; 3 Centre for Sustainable Area Management (CeSAM), University of Bergen, Bergen, Norway; 4 Department of Human Nutrition and Dietetics, College of Medicine and Health Sciences, University of Rwanda, Kigali, Rwanda; 5 Centre for the Study of Sciences and Humanities, University of Bergen, Bergen, Norway; 6 Centre for Nutrition, University of Bergen, Bergen, Norway; 7 Mohn Nutrition Research Laboratory, Department of Clinical Medicine, University of Bergen, Bergen, Norway; 8 Department of Medical Biochemistry and Pharmacology, Haukeland University Hospital, Bergen, Norway

**Keywords:** Diet, Meat, Animal-sourced foods, Protein, Sustainability, Aging, Older adults, Kigali, Rwanda, Sub-saharan Africa

## Abstract

**Background:**

The global population is increasingly aging, imposing a substantial burden on social and healthcare systems as aging is associated with gradual muscle wasting and functional decline. Consumption of protein-rich foods, such as livestock-based meat, providing high-quality proteins can prevent muscle wasting and related functional decline in older adults. However, there is a lack of data on the older adults’ perceptions about meat consumption, particularly in Sub-Saharan Africa.

**Objective:**

To explore the perceptions about dietary meat consumption among older adults in Gasabo district, Kigali, Rwanda.

**Methods:**

We conducted a descriptive qualitative study, using focus group discussions. A total of eight FGDs, lasting between 55 and 80 min, were conducted with gender-mixed groups, including 31 men and 33 women aged ≥ 55 years old. Eight older adults participated in each FGD session, and all discussions were conducted in the local language (Kinyarwanda) using a pre-designed interview guide. The discussions were audio-recorded and transcribed verbatim and translated into English. The transcript was inductively analyzed using thematic analytical process.

**Results:**

Three themes were identified from the data, predominantly related to motives and barriers of meat consumption. The motives of meat consumption included improved quality and taste of the diet, improving own health, and having a social function. Barriers of meat consumption were perceived to be related to health risks, sustainability concerns (depletion of resources), and religious beliefs. Lastly, it was widely perceived that meat was unavailable and economically inaccessible, thus meat consumption was perceived to be associated with improved wealth.

**Conclusion:**

The findings revealed a low and declining meat consumption among older adults due to poverty. Improving financial capacity or strategic public health work to improve protein consumption in the elderly is necessary to meet nutritional needs and facilitate healthy aging.

**Supplementary Information:**

The online version contains supplementary material available at 10.1186/s12889-024-19038-z.

## Background

The global population is increasingly aging, imposing a substantial burden on social and healthcare systems [1]. Globally, the number of older persons (aged over 60 years) was estimated to be around 900 million in 2015, and this number is expected to rise, reaching over 1.4 and 2 billion in 2030 and 2050 [2], respectively. Global longevity is due to the rising life expectancy (LE) and mortality decline [3], where in the past two decades, the average years of LE rose from 66 to 73 years [2]. In Africa, LE has also increased, reaching around 60 years in 2021, and it is expected to continue rising in the next decades [4]. In Rwanda, LE has similarly increased over the past years, and it was estimated to be around 70 years in 2022 [5].

Aging increases the risks of poor health and malnutrition due to physiological, physical, and psychosocial changes with advancing age [6]. Skeletal muscle wasting is one of the physiological changes that begins in midlife and gradually intensifies in later life [7]. The loss of muscle mass is partly due to inefficient synthesis of muscle protein in older people [8], resulting in a relatively higher protein requirements for the older population. The loss of muscle mass is associated with many consequences in older persons, including the loss of strength and increased functional dependency [9]. Additionally, skeletal muscles play a major role in homeostatic regulation of bodily glucose, and thus, the decline of muscle mass negatively affects glucose control [10].

Adequate protein consumption can prevent muscle wasting and related functional decline in older adults [11]. However, increasing protein intake of older persons continues to be a challenge as food intake declines with advancing age [12]. The decline in food intake is primarily related to the sub-optimal function of the digestive system and poor appetite in older persons [13]. Changes in social and eating behavior are also among the non-physiological factors that have been shown to affect food intake in later life [14]. Many older adults experience bereavement, depression, and loneliness, reducing their apathy towards food [15].

To meet the protein needs of older adults, they need to consume foods that are rich in protein, such as fish, milk, and meat [16]. These protein-rich foods provide large amounts of high-quality protein at consumption of relatively small amounts of foods [17]. Proteins from animal-sourced foods (ASFs) have also an advantage of being more bio-digestible than plant-based proteins [18]. In addition to enriching the diet, ASFs consumption has been associated with increased appetite in older adults [19]. Thus, ASFs can improve both food acceptability and protein intake in older persons.

Generally, people do not consume an isolated nutrient but rather select foods providing that particular nutrient [20]. However, food choices are complex, and food consumption motivations extend beyond physiological needs [9, 21]. Food choice process of the older adults is, particularly, heterogenous [22], and it is it influenced by different factors, including age-related chemosensory changes, personal health, and living situation [22, 23]. Moreover, prior experience with food, including self-sufficiency and hunger, have also an influence on how older people view and decide what to eat [24].

ASFs generally receives positive nutritional disposition and sensory ratings compared with plant-based foods [20, 25]. On the other hands, people also associate ASFs consumption, particularly red and processed meat, with undesirable health and environmental effects, including climate change and cardiovascular diseases [26]. However, most of the available research on adults’ behavior about ASFs consumption has been limited to high-income countries (HICs) [27]. In these countries, motives to consume or not consume meat have been perceived to be related to health, environmental sustainability, animal welfare, sensory appeal (taste, texture, flavor, etc.), costs, and socialization [12, 20, 28, 29]. In Sub-Saharan Africa, the studies investigating ASFs consumption among older adults have been limited to understanding the role these foods play in the diversity and quality of the diet [30, 31]. Thus, studies assessing older adults’ perceptions about meat consumption in sub-Saharan Africa are still lacking.

Moreover, although protein-rich foods are essential for protein needs in later life, studies, mainly from HICs, have shown that older adults may lack awareness about the importance of meat and other protein-rich foods in healthy aging [19, 25, 32]. Additionally, perceived motives and barriers of dietary meat consumption in older adults from LMICs may differ from HICs. Therefore, the aim of this study was to explore the perceptions about meat consumption in a sample of older adults (≥ 55 years old) residing in Gasabo district, Rwanda.

## Methods

An exploratory, descriptive, qualitative study design was employed to explore the perception about meat consumption among older adults, as described by Sandelowski (2000) [33]. Descriptive qualitative study design is used to explore and interpret the perceptions about a particular issue, problem, or phenomenon as experienced by the participants in a real-life context [34]. Descriptive qualitative design was also deemed appropriate for this study based on its data-driven orientation in exploring people’s perceptions and experiences [33].

### Study context

This study was conducted as part of a larger research project seeking to understand protein intake, dietary sources of protein, and nutrition status of older adults in Gasabo District, Rwanda. One part of this main project was to assess protein intake of older adults and estimate the contribution of ASFs to total protein intake using a (quantitative) dietary survey. However, quantitative studies are not appropriate for exploring beliefs and perceptions, and their results do not provide an understanding of why certain foods are eaten or not. Thus, this study was conducted to gather the viewpoints of older adults on meat consumption in Gasabo district, Kiali City, Rwanda.

Rwanda is a small, landlocked, and densely populated country with a population of about 13.5 million [35]. After the 1994 genocide against the Tutsi, the country is undergoing urbanization and economic transition [35]. The urban population in Rwanda rose from 4.6% in 1978 to 16.5% in 2012, and 28% in 2015 [35]. The country also recorded one of the fastest-growing economies in Africa, with annual gross domestic product (GDP) growth averaging over 7% between 2008 and 2017 [36].

Kigali is the capital city and largest urban settlement in Rwanda, and one of Africa’s fastest-growing cities. The estimates show that the population of Kigali city grew from around 350,000 in 1996 to over 1,7 million in 2022 [5, 37]. This rapid growth in urban settings is driven by rising life expectancy, population growth (2.9%), and rural-to-urban migration [38]. Kigali city comprises three districts, namely Kicukiro, Gasabo, and Nyarugenge district. Constituted by 15 sectors, Gasabo is the largest district in Kigali, and it hosts more than half (55.1%) of Kigali city’s residents [5].

### Selection of the study participants

In qualitative studies, the main goal of sampling is to identify people experiencing or who are closely related to the condition (context) being studied [39]. The identified people are expected to provide information-rich data required to explore the topic or phenomenon of interest [39]. It is also important that the selected people share homogenous experiences on the issue or phenomenon being studied [40]. . Variation in the sample units brings different perspectives required to capture various experiences related to the subject [41], and efforts should be taken to achieve variation.

In our study, the participants were also purposively recruited from eight sectors covering urban and peri-urban areas of the Gasabo district. The participants were enrolled in the study between January and February 2022 if they fulfilled all the following criteria: (1) were aged ≥ 55 years old, (2) were meat-eaters, (3) lived in Gasabo district for at least six months before the study, (4) were involved in food purchase or preparation within the household, and (5) able to provide informed consent. The criterion for being involved in household food purchase decisions was set to ensure that the participants have, in one way or another, experience and an understanding of the food market in Rwanda.

The recruitment process was jointly completed by the research team and community health workers (CHWs). First, the research team identified potential participants during a parallel dietary cross-sectional study conducted from November 2021 through January 2022. The CHWs used population lists retrieved from administrative unit stratified on the age criterion and they were randomly sampled. For the qualitative study, the research team asked the CHWs to identify and suggest other older adults fulfilling the eligibility criteria in their villages but not taking part in the dietary survey. The CHWs were familiar with the purpose of the study, and they were also involved in the quantitative study and served as the liaison between the research team and study participants in the dietary survey. The CHWs were trained on the specific profiles of the participants eligible to partake in FGDs. The recruitment process was conducted separately in each sector, and enrolment was closed upon reaching the desired group size (*n* = 8). While recruitment sought diverse representation and covered both urban and peri-urban areas, the selected villages constituted a convenience sample.

### Focus group discussion

We chose focus group discussion (FGD) as our method to gather ideas and opinions on meat consumption practices [42]. Previous research has shown that focus group discussions are suitable for exploring meat consumption behaviors by allowing individuals to interact freely and share ideas, opinions, and lived experiences [28]. The FGD method is particularly useful for capturing the ‘why’ and ‘how’ required to understand a specific issue or phenomenon [42].

Initially, six FGDs were planned. However, eight FGDs were conducted. Two additional FGDs were conducted to gather assertive ideas and views on some topics that emerged as important. Each FGD session lasted between 55 and 80 min. Eight participants (men and women) were invited per FGD, and the number was decided based on the recommended group size (6–12 participants) to allow for optimal interaction [43]. Mixing men and women in the same FGDs may be problematic when discussing sensitive issues, such as domestic violence and reproductive health, which affect men and women differently [44]. Failing to address such issues may lead to limited disclosure and expression of socially desirable views. Our topic of discussion was dietary meat consumption, and it was, therefore, deemed unlikely that having men and women in the same FGD would affect self-expression and discussions.

Sessions of FGDs were conducted in the villages of the participants. In Rwanda, villages are the smallest administrative units after Cells and Sectors, in ascending order. All these administrative units have venues (halls) designed for community meetings. Our FGDs were conducted either in the villages or cells’ meeting halls, depending on the availability and accessibility of the venue.

All FGDs were conducted using an interview guide (See S1 Table 1), and the interview guide’s questions were adapted from previous studies investigating meat consumption behaviors [20, 45–48]. The adaptation involved translating the questions from English to Kinyarwanda. The translated questions were also checked and rephrased by the research team to ensure semantic equivalence. All FGDs were conducted in Kinyarwanda (local language) and facilitated by the first author (TH) and a research assistant (PN), who were native speakers and experienced with qualitative methods. The focus group discussion guide included open-ended questions exploring the participants’ perceptions regarding personal views and lived experiences on meat consumption. The interview guide also contained items aimed to capture how older adults situate meat in the broader scope (theme) of health and sustainability as well as over- and under-consumption. A pilot FGD was conducted and analyzed in one sector, resulting in minor changes to the interview guide. However, the interview guide changed only in wording, and the data collected from the pilot FGD were used in this study. All FGDs were audio-recorded with the permission of the participants.

**Table 1:** Demographic characteristics of the study participants

	All ( *n* = 64)	Women (*n* = 33)	Men (31)
Age, median (IQR)^1^	64 (59–70)	64 (59–70)	65 (60–69)
Marital status, n (%)^2^			
Cohabiting/ Married	22 (50.0)	7 (26.9)	15 (83.3)
Single /divorced /Widowed	22 (50.0)	19 (73.1)	3 (16.7)
Family size, median (IQR)^3^	5 (4–6)	5 (4–6)	6 (4–7)
Education, n (%)^4^			
No education	19 (32.8)	15 (45.5)	4 (16.0)
Primary	32 (55.2)	13 (39.4)	19 (76.0)
Secondary or Higher	7 (12.1)	5 (15.2)	2 (8.0)
Wealth category, n (%)^1^			
Category 1 (Poorest)	10 (16.7)	7 (21.2)	3 (11.1)
Category 2 (Poor)	22 (36.7)	13 (39.4)	9 (33.3)
Category 3 (Rich)	27 (45.0)	13 (39.4)	14 (51.9)
Category 4 (Richest)	1 (1.7)	0 (0.0)	1 (3.7)

Abbreviation: IQR (25%, 75%), Interquartile range

^1^*n* = 61, ^2^*n* = 44, ^3^*n* = 60, and ^4^*n* = 58

Demographic characteristics of the participants we recorded were age, sex, marital status, family size, education level, and wealth category. In Rwanda, a wealth index classification system exists, and it is based on a combination of the household’s income, properties, and assets [49]. The categories range from 1 to 4, corresponding to 1 = poorest, 2 = poor, 3 = rich, and 4 = richest [49]. Focus group participants received 5000 Rwandan Francs (equivalent to 5 USD) as reimbursement for travel expenses.

### Operational definition of “meat”

The meaning of the term “meat” may vary depending on the context, and thus, different levels of characterization are used to differentiate between types of meat, including the sources (wild/bush vs. livestock-based), processing level (processed vs. unprocessed), mode of production (conventional vs. organic), religion (halala vs. haram), and environmental footprint (low vs. high-carbon intensive) of meat. The present study focuses on the role of meat in the diet as a source of animal proteins and its role in both health and sustainability. Thus, the term meat refers to livestock flesh, including beef, pork, goats, sheep, and poultry (chicken, turkey, duck, etc.). This contextualization of meat was informed by the dominance of livestock-based meat in the Rwandan diet compared with other ASFs [50]. As a landlocked country, consumption of fish and seafood is very limited in Rwanda, contributing to < 3% of daily (total) protein intake [51]. Moreover, similar to other LMICs, animal-based proteins are produced from dual-purpose livestock in Rwanda, where eggs and milk are the co-products of meat-producing ruminants (beef, goat, and sheep) and non-ruminant (poultry) livestock [52].

### Data processing, synthesis, and interpretation

All FGDs were transcribed verbatim, and transcripts were checked and enriched with field notes. The first author and a professional translator independently translated the transcript from Kinyarwanda to English. Two translated copies were compared, and discrepancies were discussed to generate the final transcript.

Synthesis of the data was completed using a thematic analytic process [53]. Thematic is a flexible analytical process that is employed to synthesize large amounts of text-based data, answering an exploratory qualitative question [54]. This analysis process is particularly useful in exploring people’s views, opinions, knowledge, or experiences on a specific subject [53]. Thematic synthesis of the data can be done either inductively or deductively. Deductive synthesis deploys a pre-designed theoretical framework or coding scheme [55]. In contrast, inductive thematic synthesis is a flexible and data-driven analytical process where the researchers iteratively code and reflect on the data to identify meaning, patterns, and themes [55]. Our study was exploratory in nature, and therefore, the transcript was inductively analyzed using the open coding process. Thematic analysis has also been widely applied in studies analyzing focus group discussions exploring meat consumption behaviors [9, 45].

Our thematic analysis followed the approach described by Braun & Clarke (2006) [56], where the transcript was first read thoroughly to familiarize with the data, followed by coding. The coding process started with generating keywords or phrases that best described the participants’ accounts. The draft of codes was shared and discussed between authors. After the initial coding, the transcript was re-read thoroughly to identify new (missing) keywords (phrases) or modify existing ones. Then, all the codes generated were re-shared and discussed between authors. All similar codes were then organized into categories and compared with the initial questions in the interview guide. At this stage, only the codes fitting within the overall aim of the study were selected and used to develop categories (sub-themes) and themes that were also shared and discussed between the authors.

The analytical process was completed with the aid of Nvivo software (Version 12). This software helps to manage and easily navigate through the text of the transcript, including coding, code organization, and creation of a thematic map. Specifically, the node features of this software allowed us to collect and mark references (quote, sentence, or part of the quote) directly from participants’ account while reading the transcript. The software also helped us to organize similar references under the same node. The software contains different levels of (parent and child) nodes, and this feature also allows for creation of hierarchies linking codes to categories (sub-themes) and themes.

### Reflexivity

The first author (TH) is a nutritionist from Rwanda and holds a MSc in nutrition and health, with a particular interest in nutrition and aging as well as sustainable consumption. He was enrolled in PhD training disembarking on this topic and led the conceptualization and planning of the current research. The study data was conceptualized and analyzed in an interdisciplinary team (TH, IMSE, MK, IEM, EM, and JD) of authors with different professional backgrounds, including health, nutrition, biology, sustainability, and ethics. Whilst TH’s understanding of the Rwandan and study context was necessary and informed the planned and conducted data capture, his research interest may have also influenced the interpretation and discussion. The international collaboration has enriched the thematic analysis process and self-reflexive awareness.

The use of computer software (Nvivo) helped to sort and manage a large amount of text-based data efficiently. It also made it easy to access references within the nodes with a direct link to the part of the transcript (participants’ account) where they were derived from, and this facilitated the process of re-coding or decoding, which, in turn, increased the reliability of the analysis process. However, using computer software has some advantages in qualitative research. These tools reduce the time that researchers need to interact, reflect, and make sense of the data [57]. In our study, the first author (TH) was very familiar with the data since he was involved in the entire process, from conceptualization to data collection, transcription, translation, and data synthesis. Other authors also familiarized themselves with the data through study visits (IE) and by reading the transcript thoroughly.

### Ethical consideration

The study was reviewed and approved by the Institutional Review Board (IRB) at the College of Medicine and Health Sciences (CMHS), University of Rwanda (Ref. No: 291/CMHS IRB/2021) and the Regional Committees for Medical and Health Research Ethics of Western Norway (Ref. No: 163,823). Permission to conduct the study was also obtained from the authorities of Gasabo district (Ref. No: 1999/070102/2021). The purpose of the study was explained to all the participants, and all provided informed consent.

### Privacy and confidentiality

The privacy of the participants and confidentiality of the provided information were safeguarded throughout the study. First, after informed consent, the participants agreed to the rules for the group including respect and confidentiality. Recording of the FGDs was anonymized during data collection. This was done by giving the participants identification numbers which they used every time they responded to the questions or intervened during the discussions. This ensured that participants could not be identified during transcription, translation, and data synthesis. The audio recordings, transcripts, and demographic data were stored on the digital infrastructure offered by the University of Bergen for safe handling of personal and health data. Additionally, access to the data was only restricted to the authorized members of the research team.

## Results

A total of 64 older adults (31 men and 33 women) participated in FGDs (Table 1). Ages ranged from 55 to 91 years (median: 64 years). Their education level varied, with 32% (*n* = 19) having no education at all, 55% (*n* = 32) having primary education, and 12% (*n* = 7) having secondary or higher education. The family size of the participants ranged from 1 to 15 household members (median: 5). More than half (53%, *n* = 32) of the participants reported being classified in the poorest- or poor wealth categories, whereas 47% (*n* = 28) were classified in the rich or richest wealth category.

### Emerging themes

**Fig. 1:**
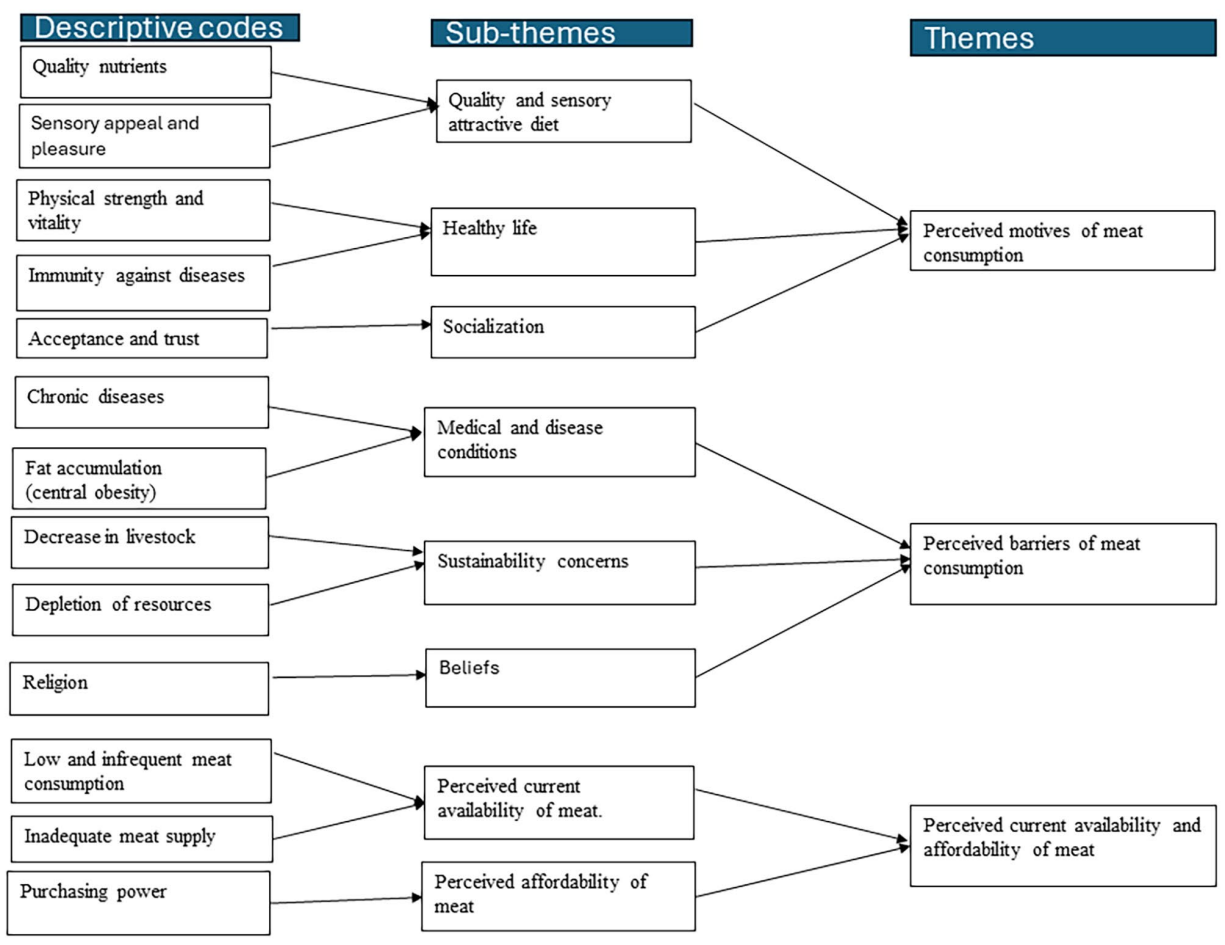
Overview of sub-themes and themes

Three main themes were identified throughout the analysis, including (1) perceived motives of meat consumption, (2) perceived barriers of meat consumption, and (3) perceived current availability and affordability of meat, Fig. 1. Example of selected quotes and related categories and themes are presented in S1 Table 2.

#### Perceived motives of meat consumption

Three categories were identified under this theme comprising diet quality and sensory attractiveness, healthy life, and socialization.

#### Quality and sensory attractive diet

The older adults’ motivation to consume meat was related to higher nutritional values and better sensory properties of meat than other (plant-based) foods. Meat was perceived as superior food, and it was explained that meat had the ability to complement nutrients lacking in plant-based foods.

*“… despite containing some nutrients, plant-based foods still need to be complemented by animal foods” FGD8, R5, Female.*

Affinity for meat consumption was also perceived to be related to the lack of alternative sources of high-quality nutrients. While elaborating on this subject, the participants described that older adults do not like to consume small animal foods like eggs and small fish. Fish (normal size, not small fish) was another source of protein that people could eat, but it was perceived not to be affordable compared to meat.

*Older people are not interested in small fish. But fish consumption is only for rich people because it [fish] is expensive. If you stop eating meat, you will likely develop deficiencies” FGD6, R8, Female.*

In addition to its nutrient content, organoleptic properties of meat were perceived to be more sensory appealing than other (plant-based) foods. The participants described that adding meat to one’s diet improved the taste of foods and made it more presentable (attractive). But, most importantly, these attributes made older adults associate meat consumption with eating pleasure and a positive psychological feeling.

*“When you eat meat, it also gives you the appetite to eat other foods. You feel energized and feel good … Your body feels much more relaxed. It makes you happy and think positively” FGD7, R6, Male.*

#### Healthy life

The participants perceived that meat consumption was necessary for better health, and many of them believed that lacking meat consumption was associated with frequent episodes of illness. Nutrient deficiencies were described to be one of the points of concern for the older adults who lack access to ASFs. It was also perceived that older adults, in particular, needed to consume meat to stay healthy and physically fit. This belief in the health-promoting effects of meat would in its absence have detrimental effects including premature death.

*“… not eating ASFs [meat] can lead to diseases or dying prematurely. It may result in the loss of strength for older people, and frequent sickness due to the lack of meat’s nutrients” FGD8, R1, Female.*

#### Socialization

Meat consumption was perceived to be essential for the family relationships and social networks of the participants. It was described that people would be judged negatively by their friends and families if they decided to reduce or stop eating meat. The role of meat consumption in social and family life was also viewed (framed) in the lenses of enhanced trust, bonding, and social status.

*“. If they see that I have stopped eating meat while I used to eat it [meat] with them, they will start saying that she no longer has money to buy meat. They will say that she has become poor and devastated” FGD2, R5, Female.*

It was explained that people would be perceived to distance themselves from their peers or have changed their socio-economic status if they stopped sharing meat with others, and thus, meat served as an ingredient in building and keeping social relationship.

*“If you do not eat meat, they will say that you are becoming greedy, or it is because you have become rich and you are no longer in their peer category” FGD7, R1, Female.*

#### Perceived barriers of meat consumption

Just as beliefs about health and diseases influenced the positive attitudes, meat was also believed to be negatively related to health and sustainability. Religious beliefs could also result in meat avoidance.

#### Medical and diseases conditions

Medical and diseases conditions were perceived to inhibit people from consuming meat. Some of the participants explained that they or their peers received medical recommendations advising them to reduce or stop meat consumption. Most of the conditions preventing them from consuming meat were allergies and underlying chronic disease conditions, such as gout, hypertension, and type 2 diabetes. High or excess meat consumption was not favored, and it was perceived that it could lead to adiposity and distorted body shape.

*“ I was also about to say that if we continue eating too much meat, … That’s what causes diseases like hypertension and types 2 diabetes. Normally, if you have those diseases, you are not allowed to eat meat. Therefore, I think that if we stop eating a lot of meat, or if we reduce eating them, those diseases will also decrease” FGD4, R4, Female.*

#### Sustainability concern

High meat consumption was perceived to be unsustainable. The unsustainability of meat consumption was discussed using its implications for the future: everybody was fully aware of its negative impact on livestock, the household’s economy, and other resources. This was highlighted by the participants’ fear of being unable to feed themselves and future generation if people continue to consume meat excessively.

“*For me, I feel that eating a lot of meat would deplete livestock, that’s the first. The second point is that if everyone eats too much meat regularly, this may affect the economy since meat is expensive. So, money-wise, you feel some pressure” FGD8. R7, Male.*

#### Religious beliefs

Religious belief was known to affect meat consumption. The Seventh Day Adventist Church members were described as the religious who reduced or avoided meat consumption.

*“ … There are other people who do not eat meat due to their religious beliefs …” FGD7, R2, Male.*

#### Perceived current availability and affordability of meat

The last theme was largely centered around low- and reduced meat consumption coupled with low affordability.

#### Perceived current meat availability

The reduced consumption was largely explained by reduced availability due to reduced production of ASFs, population growth, and changed social norms. Throughout the discussions, the participants described that both quantity and frequency of meat consumption had declined compared with the past. The decline or scarcity of meat consumption was perceived to be linked to the imbalance between the demand and supply of meat in the country. While elaborating on this topic, the participants explained that the population has increased, but the population growth was not proportional to meat production. They also explained that, besides the increasing number of people consuming meat, population growth had also affected meat availability and other ASFs by reducing arable land for livestock production.

*“ We used to eat a lot of meat and other animal products in the past because they were available. One could drink a jug of milk alone! Nowadays, a similar jug is shared at least between four people since the number of people has increased” FGD4, R7, Female.*

Moreover, from a production point of view, it was perceived, to some extent, that a change in meat production policy contributed to the decline in meat availability by reducing the number of livestock for the farmers across the country.

*“Meat used to be available in the past because farmers had enough cows [herds] grazing on hills. These days, livestock are only kept on farms, and farmers can only rear a few livestock” FGD4, R3, Male.*

The increase in the number of meat consumers was also perceived to be related to the vanishing of some social norms that previously inhibited some population groups from consuming meat and other animal products. The participants explained that, in the past, households’ meat consumption was primarily determined by men’s decisions, and women were expected to consume some types of meat only. Today, these social norms are not that evident, and women make their own decisions and can eat meat anywhere and at any time without requiring men’s approval.

*“In the past, there were few people, and there was also respect. But nowadays, I can go out with my wife, and she can decide to buy herself a brochette [grilled meat]” FGD4, R7, Male.*

#### Perceive affordability of meat

Meat was perceived unaffordable compared to other foods, and low purchasing power was frequently cited by the participants within and across the FGDs. However, the influence of purchasing power was described using high prices of meat and large families that people need to feed. The affordability of livestock-based meat was discussed by comparing it with plant-based foods and other non-livestock sources of proteins (fish and seafood). Across all FGDs, there was a consensus that livestock-based meat was more expensive than plant-based protein sources (beans, peas, lentils, etc.) while being cheaper than fish (normal sized fish).

“*… when you go to the market you buy according to your [financial] capacity and family size. You buy foods which will last longer … that’s why people buy beans instead of meat” FGD6, R2, Male.*

The perceived role of purchasing power in meat consumption was also reinforced by the view that people increase their meat consumption as their financial capacity increases.

*“ I will talk about what I see in Rwanda. When a person’s economic situation improves, a person starts eating a lot of meat …. [laughing]. Meat is always on their plate because they have that [financial] capacity” FGD6, R2, Male.*

## Discussion

This study explored the perceptions about dietary meat consumption among the older adult population (aged > 55 years old) living in Gasabo district, Kigali, Rwanda. Meat consumption was strongly related to the perceptions about diet quality and health with strong sensory and social attachment. However, it was perceived that meat consumption of older adults had declined compared with the past decades. Sustainability concern, medical and disease conditions (such as hypertension, types 2 diabetes, etc.), and religious beliefs were perceived to be the barriers of meat consumption among older adults. The perceptions about meat consumption were shared across the geographical locations (urban and peri-urban) as well as for men and women.

Meat consumption was perceived to be required for the physiological needs of older adults. The participants appeared to be aware that nutrients from meat may contribute to maintaining ‘physical strength’ and boosting the ‘immune system’. It has also been reported in other studies that perceived healthiness, in terms of nutrient content, influences older adults’ choice of meat [58]. In a study from Senegal, a preference for bush meat based was connected to its nutritional value and low price [59]. In a study from Nairobi, it was found that low-income households reported that nutritional value was the main reason to consume beef meat and other animal products (eggs, fish, and milk) [60]. In a study from Uruguay it was seen that high nutritional value motivated consumers to choose meat over other foods [61].

Besides enriching the diet, organoleptic properties of meat were perceived as important in increasing appetite. Sensory attributes have been shown to influence consumers’ preferences for animal foods over plant-based foods [20, 62]. Studies in Brazil and Switzerland have reported that sensory properties influenced meat preferences among older adults and elderlies [63, 64]. A study on barriers and facilitators of animal-based protein-rich foods found that taste was an independent predictor for the consumption of animal-based proteins [12]. An intervention study to improve intake of protein-rich foods showed that older adults needed spices to increase the taste of plant-based foods to the level of meat-based meals [65]. Additionally, the role of animal foods on food-liking has been shown by the studies mixing animal foods with plant-based foods to enhance the texture and taste of plant-based foods [66].

Older adults perceived meat consumption as essential for their social life. These findings re-affirmed that, unlike other foods, meat is valued beyond nutritional needs and taste [67]. Meat holds essential social values in various societies and is eaten for pleasure and socialization [68]. In Rwanda and other African countries, meat also plays a similar role. For example, Rwandans eat grilled meat, called Nyamachoma, together with alcohol to socialize in private and social settings [69]. In the Central African Republic of Congo, forest foragers reported using meat exchange to strengthen their relationships [70]. In the Republic of Congo, consumers ate animal foods because they were luxury items and a symbol of high social status [71]. Also in other parts of the world, similar findings have been observed, for example in New Zealand, consumers reported eating meat to socialize and feel accepted by their peers [72].

In our study, it was mostly perceived that meat consumption had declined, and it was broadly interpreted to be related to the imbalance between meat demand and supply in the country. The Rwandan population has grown exponentially over the past decades [5], and this may increase the demand for meat [73]. Population growth has been reported to affect meat availability in Nairobi, where annual meat consumption increased by 2.2% between 1980 and 2000 [74], but during the same period, per capita meat consumption decreased by 11% [74].

Change in meat consumption was also perceived to be linked with the transformation of social norms vis-à-vis the food-based myth and taboos [75]. In the past, it was culturally taboo for women to eat goat meat, and it was stereotyped that women would grow beards [76]. But, all these negative norms vanished as in the country, alongside global trends, promoted gender equality was widely [77]. On the other hand, the participants also explained that eating certain types of meat, such as rabbit and sheep, was unpopular. Many Rwandans considered sheep unclean, and it was consumed by the population in the low socioeconomic category [78]. This expansion of meat consumption to unpopular sources can also be seen as an adaptation to the increasing scarcity of meat.

Our results also revealed that only a minority of the population voluntarily reduce or stop meat consumption due to their religious belief. Contrary to religion, sustainability concern, and medical constraints were perceived as involuntary barriers for older adults seeking to consume meat. In high-income countries, there are also barriers to meat consumption in older adults which include the cost of meat, diseases (medical constraints such as oral or mental health conditions), and accessibility (e.g. distance to shopping centers) [25, 28, 79]. However, although there were concerns that it would not be enough in the future if people over-consumed ASFs, the older adults did not elaborate on choices based on climate change and animal welfare. The older adults appeared to mainly be concerned with having enough resources to cover their current dietary needs and future generations. A similar lack of awareness of the link between the ASFs consumption and animal welfare and environmental sustainability (climate change) has been reported among emerging adults in Ghana [80]. This contrasts with a growing number of consumers in HICs who are increasingly adopting meatless diets for ethical and environmental reasons [29].

Furthermore, affordability was perceived to be a major challenge for older adults seeking to consume meat and other animal products. Unlike diseases, affordability is a factor that can be modified to improve older adults’ access to meat by either targeting prices or increasing the purchasing power [81]. ASFs are highly priced foods in LMICs and tend to cost more than other staple (plant-based) foods [27]. Wealth has also been shown to positively correlate with meat consumption [82]. The relationship between wealth and meat consumption was shown to be stronger in the population with a rising economy than in the affluent population [83]. This may also imply that targeting poverty reduction may increase access to meat consumption in the older adult population. It also highlights that there are opportunities to influence Rwandan consumers to make conscious demand for meat.

Moreover, based on the Rwanda’s economic growth recorded in the last decades, one would expect a perceived increase in the affordability of meat, and thus, an increased meat consumption. But it is possible that the income growth have not reached the threshold needed to make dietary shifts toward meat-rich diets [84]. Of note, this study was conducted on a sample of predominantly older adults in the poorest wealth categories (53%), and whose income may not increase [85]. Further, this study was conducted during the Covid-19 pandemic that affected families’ economy and income, and this may also have influenced how older adults viewed highly priced food products, including meat [84].

### Strengths and limitations of the study

This study employed a focus group discussion (FGD) method, allowing the participants to interact freely. The data from FGDs enables the analysis and interpretation of the social meaning of the views and opinions expressed on the studied topic [42]. The study team iteratively reflected on data and assumptions made during study design, enhancing methodological rigor. However, the data presented in this study are solely based on public (community) perspectives as no additional data collection method or informant groups were included, such as in-depth interviews or key-informant interviews with leaders, health workers, and other relevant groups. As the participants were from the capital city of Rwanda, their views cannot be generalized to the population in rural settings. Further, the study only included older adults who are meat eaters by preference, and their views may also not entirely represent the views of the population who do not eat meat for various reasons other than religion, income, and disease conditions. Men and women were mixed in the groups which we assumed would stimulate the discussion, and we cannot rule out any power, social and gender dynamics which may have affected the responses. Using CHWs as recruiters, there is always a risk of preferential selection, although we believe that necessary training and follow-up was done to mitigate that. Moreover, this study only highlighted the factors perceived to influence meat consumption among older adults, but their relative importance remains unknown. Thus, future behavioral studies are also needed to identify the most important factors influencing meat consumption in this population.

### Implications for research and policy

Despite the limitations, the findings of this study provide an important contribution with relevant implications for both research and policy. First, the findings of this study provide an empirical foundation for future investigations of dietary behavior about ASFs consumption, meat in particular, among older adults in Rwanda, but with assumed relevance to other Sub-Saharan African countries. From policy perspectives, motives and barriers to meat consumption identified in this study can inform the development of strategies to increase access to protein-rich foods and promote healthy aging in Rwanda. This is important, considering that older adults are becoming a large part of societies both in both low- and high-income settings and also given that older persons are disproportionally affected by poor health and malnutrition, including muscle wasting.

## Conclusion

Older adults perceived that meat consumption had declined over time compared with the past decades. The study also revealed motives and barriers influencing meat consumption among older adults rising the capital city of Rwanda. Overall, our study included a high percentage of persons in the lower wealth categories and thus highlighted that improving financial capacity can be targeted by interventions seeking to increase protein consumption among the older adult population with inadequate meat consumption. However, population studies using different designs and methodologies, including older adults from rural and urban settings, are needed to fully understand current practices among older adults in Rwanda.

### Electronic supplementary material

Below is the link to the electronic supplementary material.

Supplementary Material 1

## Data Availability

The data that support the findings of this study are available from the corresponding author (TH) up a reasonable request.
