# Supplementary material for: Older adults’ perceptions about meat consumption: a qualitative study in Gasabo district, Kigali, Rwanda
Source: BMC Public Health. 2024 Jun 5;24:1515. doi: 10.1186/s12889-024-19038-z (PMC11155052; doi:10.1186/s12889-024-19038-z)
Supplement: Supplementary file 1 — Supplementary Material 1 [file 12889_2024_19038_MOESM1_ESM.pdf]

S1 Table 1. Interview guide for FGDs

	**Point of discussion (questioning route)**
Introductory questions	What does meat consumption mean to you (in your daily life)? Any association?
Transition questions	In your view, what are the trends in meat consumption over the past decades in Rwanda? (Are there any changes in meat consumption patterns over the past few decades?)
	Are there changes in the environment and climate you see here in Rwanda?
Core questions	What do you think about eating less meat (animal foods) than we eat nowadays?
	How would the most important people in your life (best friends, colleagues, family, or relatives) react if you decided to reduce or quit meat (animal foods) consumption?
	Do you think that eating a lot of meat (animal foods) has caused, is causing, or will cause any problem to the personal health, climate, or the environment?
	Do you think that reducing or avoiding meat (animal foods) consumption may cause any problems or has any consequences on the environment, climate, and human health?
Ending	What do you think about the affordability of meat compared to other foods in Rwanda?
	Do you have any remarks, suggestions, or additions?

S1 Table 2. Examples selected quotes, and related categories and themes

**Theme 1: Perceived motives of meat consumption**		
Quality and sensory attractive diet	Quality nutrients	*"If a person does not eat meat… that's when you start seeing that person becoming shaky. This is because, despite containing some nutrients, plant-based foods still need to be complemented by animal foods" FGD8, R5, Female.*<br><br>*"What does eating meat mean to me? Like what other people said, meat provides nutrients to the body" FGD5, R3, Female.*<br><br>*"I eat meat at least once per week … You cannot get money to eat meat every day. But, in general, meat is good and nutritious" FGD8, R2, Female.*<br><br>*Older people are not interested in small fishes. But fish consumption is only for rich people because it [fish] is expensive. If you stop eating meat, then you are likely to develop deficiencies" FGD6, R8, Female.*
	Sensory appeal and pleasure	*"The reason why I eat meat is because it gives my appetite to eat other foods. If you eat well-cooked meat, it increases your appetite to eat other foods" FG7, R2, Male.*<br><br>*" …If you prepare meat and meat stew, the food [dish] looks attractive and tasty […]" FGD6, R2,Male.*<br><br>*"I want to add something! I eat meat because I enjoy eating it. Whatever food I eat with meat, either Irish potatoes, rice, banana, or cassava dough, it goes well. I eat meat because it is tasty, but otherwise, I have no issue with eating other [plant-based] foods" FGD6, R5, Male.*<br><br>*"When you eat non-meat containing foods/meals only, it arrives to the point where you lose appetite … FGD7, R1, Female.*<br><br>*"…when I go some days without eating them[meat], I get some strange feeling in my stomach. Eating beans? No! Eating vegetables? No! Only eating meat makes me feel good" FGD8, R2, Female.'*

		“If I manage to buy 2 kilograms of meat, cook it for the children, and they are happy if each of them eats two or three pieces ….” FGD5, R6, Female.<br><br>*“When you eat meat, it also gives you appetite to eat other foods. You feel energized, and your body feels good. But most importantly you feel like there is a difference in your body where you become much more relaxed. It makes you think positively as well as feeling happy” FGD7, R6, Male.*
Healthy life	Physical strength and vitality	*“It's really good to eat meat … it [eating meat] makes bones stronger and gives you energy... “ FGD6, R4, Female.*<br><br>*"… not eating ASFs [meat] can lead to diseases or dying prematurely. It may result in the loss of strength for older people, and frequent sickness due to the lack of nutrients" FGD8, R1, Female.*<br><br>*“Eating meat provides energy and strengthens bones. For us who are farmers, once you have eaten meat, you feel strong and ready to carry out faming activities. I usually eat meat when I feel like I’m missing them. When I’m sick, I eat meat and feel re-energized” FGD7, R3, Female.*<br><br>*“I feel that eating less meat or quitting (lacking them) them [meat] completely is like inviting disability or disease in your courtyard" FGD8, R5, Female.*<br><br>*“… the body needs them [meat]… we know that meat builds the body. We need to build our bodies, especially for us who are old. If we do not eat them our bodies might become weaker” FGD6, R8, Female.*<br><br>*“ Not eating meat makes you unhealthy because all animal foods are important in our bodies. You should at least drink milk if there is no meat [if you can’t eat meat]. But if you do not eat any of them (lack both), then you are in troubles” FGD1, R2, Female.*<br><br>*“Meat is good for health because it increases the blood, especially when it is eaten as plain meat stew without oil. You eat it and sweat; you feel heat. It makes blood circulate well in the body” FGD6, R6, Male.*

	Immunity against diseases	*“When you eat meat, you become stronger. Eating meat makes you feel better if you were having something like flu” FGD3, R4, Male.*<br><br>*“Sometimes you feel sick, and you start thinking that it's because you haven't eaten meat in a while. Then, you decide to ignore buying other foods and spend the money on meat because you feel that the body is missing something" FGD5, R4, Male.*
Socialization	Acceptance and trust	*“For a single mother and breadwinner like me, deciding to quit or reduce meat consumption would make your children think that you are doing it so that you’ll no longer buy meat for them” FGD6, R8, Female.*<br><br>*“If I decide to quit meat, the people who I usually eat (share) them with, including family member or colleagues, will feel embarrassed. They will say that you are becoming a greedy person, or it is because you have become rich, and you are no longer in their peer category” FGD7, R1, Female.*<br><br>*" If I decide to reduce or stop eating meat, my family and friends won’t believe it. They will say that I eat meat in bars or restaurants. Even if I continue to buy a lot of meat for them [family member], they won't believe it. They will still say that I eat meat when I go out. They will start thinking that I have become greedy and selfish" FGD2, R6, Male.*<br><br>*“.. If they see that I have stopped eating meat while I used to eat it [meat] with them, they will start saying that she no longer has money to buy meat. She has become poor and devastated” FGD2, R5, Female.*
**Theme 2: Perceived barriers of meat consumption**		
Medical and disease conditions	Chronic diseases	*“Sometimes you become sick. Then, when you go to the hospital, they stop you from eating meat depending on the disease you are diagnosed with, FGD4, R1, Female.*<br><br>*“… when I eat meat, I get allergic reactions. When I wake up, my eyes are swollen ….” FGD5, R6, Female.*<br><br>*“ I was also about to say that if we continue eating too much meat, … That’s what causes diseases like hypertension and types 2 diabetes. Normally, if you have those diseases, you are not allowed to eat meat. Therefore, I think that if we stop eating a lot of meat, or if we reduce eating them, those disease will also decrease” FGD4, R4, Female.*

		"… You develop disease like gout! If a person has eaten a lot of meat, this leaves a lot of waste in the body. This waste blocks vessels, and a person develops gout. Do you see? That is problematic" FGD5, R1, Male.<br><br>*"… Meat causes gout. To be honest, I also suffer from gout, but I wasn't eating meat. I used only to eat goat meat, and then I developed gout […]" FGD8, R8, Male.*
	fat accumulation (central obesity)	*“Eating meat is good but if you eat too much you are destroying your body. Some people eat brochette [grilled meat] and eat them not well cooked or roasted. Then, you find a man becoming as if he is pregnant" FGD6, R6, Female.*<br><br>*"When you eat a lot of meat, and eat them consecutively, there is no doubt that there will be consequences. One sometimes develops a big belly…." FGD4, R8, Female, .*
Sustainability concerns	Decrease in livestock	*"I think we should eat less meat and other animal products because we (people) are increasing. If you eat a lot, you will not be able to feed yourself. You need to ensure that this one [referring to a child sitting around] will also be able to get enough. Let's assume that you use three liters of milk daily and have three children. Do you think you will be able to continue to supply that amount?" FGD4. R7, Female.*<br><br>*“ Eating a lot of meat may cause a problem! The livestock are decreasing because we eat them. For instance, you can eat meat, and eat meat every day. If you had chicken, rabbit, or sheep, and they gradually decrease because they have been constantly eaten” FGD3. R7, Female.*<br><br>*“ There may be some consequences if people continue to eat a lot of meat. The consequences would happen to the livestock as they may vanish. Livestock would not increase because they are being eaten. In contrast, there may be no consequences on milk and eggs, because these are co-products of livestock” FGD3, R4, Male.*
	Depletion of resources	*"Eating too much meat negatively affects economic situation at home because meat is expensive …" FGD7, R2, Male.*<br>“*For me, I feel that eating a lot of meat would deplete* ***livestock****, that’s the first. The second point is that if everyone eats too much meat regularly, this may affect the economy since meat is expensive. So, money-wise, you feel some pressure” FGD8. R7, Male.*
Religious beliefs	Religion	*“Due to religious beliefs, seventh day Adventists don't eat meat. They use soyabeans to make tofu. That's what they replace meat” FG6, R2, Male.*<br><br>*" … There are other people who do not eat meat due to their religious beliefs …" FGD7, R2, Male.*

**Theme 3: Perceived current availability and affordability of meat**		
Perceived current availability of meat.	Low and infrequent meat consumption	*“ We used to eat a lot of meat and other animal products in the past because there were available. One could drink a jug of milk alone! Nowadays, a similar jug is shared at least between four people since the number of people has increased” FGD4, R7, Female.*<br><br>*"It was easy to get meat in the past, but now there are people who rarely eat meat because meat has decreased and it is more expensive" FGD4, R8, Female.*<br><br>*“The difference now is that, in the past, you would take a basket with you whenever you went to buy meat. That basket would be filled with meat, and you would eat it [meat] every day….]. Nowadays, a budget of 5000 – 6000 Rwandan francs (5-6 USD) buys only two kg of meat, and this is not sufficient for the family” FGD7, R4, Male.*
	Inadequate meat supply	*“Meat used to be available in the past because farmers had enough cows [herds] grazing on hills. These days, livestock are only kept on farms, and farmers can only rear a few livestock" FGD4, R3, Male.*<br><br>*“Our country is undergoing development, and the population has increased. Wherever there were farms, they replaced them with buildings. You can't find any space to rear cattle. The size of farms [cultivable land] has been reduced compared to the number of people who need to be fed..." FGD7, R1, Female.*<br><br>*"In the past, there were few people, and there was also respect. But nowadays I can go out with my wife, and she can decide to buy herself a brochette [grilled meat]" FGD4, R7, Male.*<br><br>*"Meat used to be available in the past because farmers had enough grazing land, but now there is a zero-grazing policy. So, livestock have become fewer, resulting in a limited availability of meat" FGD4, R3, Male.*
Perceived affordability of meat	Purchasing power	"*… when you go to the market you buy according to your [financial] capacity and family size. You buy foods which will last longer, regardless of which nutrients they will obtain. That’s why people buy beans instead of meat” FGD6, R2, Male.*<br><br>*"People who eat a lot of meat, it is because they can afford it … they have resources" FGD3, R5, Male.*<br><br>*People do not eat meat because they do not have resources to buy it [meat]. You cannot go to buy one kilo (kg) of meat for 3500 Rwandan francs (3.5 USD) if you do not have money to buy even one kilo of beans. That's why a person can go for a year without eating meat” FGD7, R7, Male.*

		"*It's also because of poverty, otherwise none would stop eating meat. Those who eat a lot of meat it is because they have (financial) resources" FGD5, R1, Male.*<br><br>*"People who eat a lot of meat it is because they have (financial) capacity. Those who eat less meat it is because they lack (financial) capacity …" FGD8, R5, Male.*<br><br>*" … we eat beans and cassava because people don't have money, we can't eat it [meat] like beans or cassava. There are those people who eat meat only when it's a feast or special day. These people normally do not eat meat due to the lack of money. But they plan that they will be eating meat on that special day" FGD2, R8, Female.*<br><br>*"I eat less meat because I have a big family that I feed. We cannot eat a lot of meat while we do not even find enough meat for us. I can quit them if I have a disease that prevents me from eating them" FGD7, R1, Female.*
